# Mitochondrial RNA in Inflammation

**DOI:** 10.7150/ijbs.119841

**Published:** 2025-08-22

**Authors:** Jian Chen, Chen You, Haibo Xie, Qixing Zhu

**Affiliations:** 1 Department of Dermatology, the First Affiliated Hospital of Anhui Medical University, Hefei, Anhui, China.; 2 Key Laboratory of Dermatology (Anhui Medical University), Ministry of Education, Hefei, Anhui, China.; 3 Institute of Dermatology, Anhui Medical University, Hefei 230032, Anhui, China.; 4 Department of Occupational Health and Environmental Health, School of Public Health, Anhui Medical University, Hefei, Anhui, China.

**Keywords:** Mitochondrial, Inflammation, mtRNA, mt-dsRNA.

## Abstract

Mitochondria are dynamic organelles integral to cellular energy metabolism and homeostasis. Beyond their traditional roles, a growing body of evidence underscores the importance of mitochondria as pivotal regulators of innate immune signaling pathways. Recently, mitochondrial RNA (mtRNA) has been identified as a novel modulator of inflammatory responses. mtRNA is detected by intracellular pattern recognition receptors (PRRs), which subsequently activate the mitochondrial antiviral-signaling protein (MAVS) and the interferon regulatory factor 3 (IRF3)/nuclear factor kappa-light-chain-enhancer of activated B cells (NF-κB) signaling axis, as well as inflammasome pathways. This activation leads to the production of type I interferons and pro-inflammatory cytokines. Furthermore, mtRNA facilitates the propagation of inflammatory signals through exosome-mediated intercellular transfer. Among the various forms of mtRNA, mitochondrial double-stranded RNA (mt-dsRNA) is particularly prone to activating inflammatory responses due to its distinctive double-helical structure. The aberrant accumulation of mt-dsRNA is strongly linked autoimmune diseases, degenerative disease, Liver Disease, kidney disease, cancers, cardiovascular diseases, and respiratory ailments. This review proposes innovative therapeutic strategies aimed at degrading pathological mtRNA or interrupting inflammatory pathways by targeting critical regulatory nodes in mtRNA metabolism and its downstream inflammatory processes.

## 1. Introduction

Mitochondria, semiautonomous organelles in eukaryotic cells, are characterized by a double-membrane system, comprising the outer mitochondrial membrane (OMM), inner mito-chondrial membrane (IMM), and mitochondrial matrix. Their dynamic network properties maintained through the fusion/fission balance are crucial for cellular energy homeostasis 1, 2. Mitochondria are the energy production centers of cells, participating in processes such as oxidative phosphorylation, ATP synthesis, fatty acid oxidation and decomposition, as well as the synthesis of lipids and heme. Moreover, mitochondria are involved in the regulation of the intracellular calcium ion (Ca^2+^) signaling transduction and modulate caspase activation and cell death through the release of cytochrome c 3-5. Numerous studies have demonstrated that under stress conditions, mitochondrial DNA (mtDNA) released into the cytoplasm functions as a damage-associated molecular pattern (DAMP) 6. Furthermore, extensive research has demonstrated the pivotal role of mitochondria in innate immunity, with mitochondrial RNA (mtRNA) emerging as a novel immunomodulatory molecule critically involved in inflammatory responses 7, 8. During cellular stress or injury, mtRNA can leak into the cytoplasm or extracellular environment through multiple pathways, functioning as DAMPs that activate pattern recognition receptors (PRRs). This interaction triggers inflammatory cascades through innate immune pathways 9, 10.

At disease level, mtRNA-mediated innate immune activation shows significant associations with various inflammatory pathologies. In osteoarthritis, extracellular mtRNA activates protein kinase R (PKR) and toll-like receptor 3 (TLR3), promoting proinflammatory cytokines production and apoptosis, ultimately leading to chondrocyte degeneration and joint deterioration 11. Systemic lupus erythematosus (SLE) exhibits increased mtRNA release, which activates inflammatory responses through cytoplasmic PRRs including retinoic acid-inducible gene I (RIG-I), melanoma differentiation-associated protein 5 (MDA5), and toll-like receptor 7 (TLR7) 12.

Investigating the metabolic regulation of mtRNA and its interaction with inflammatory processes offers significant insights into the pathogenesis of inflammatory disorders. Furthermore, it aids in the development of targeted therapeutic strategies. These advancements are promising for the progression of precision medicine in diseases associated with inflammation.

## 2. Structure and Metabolism of mtRNA

### 2.1 Transcription and Processing of mtRNA

**Figure 1:**
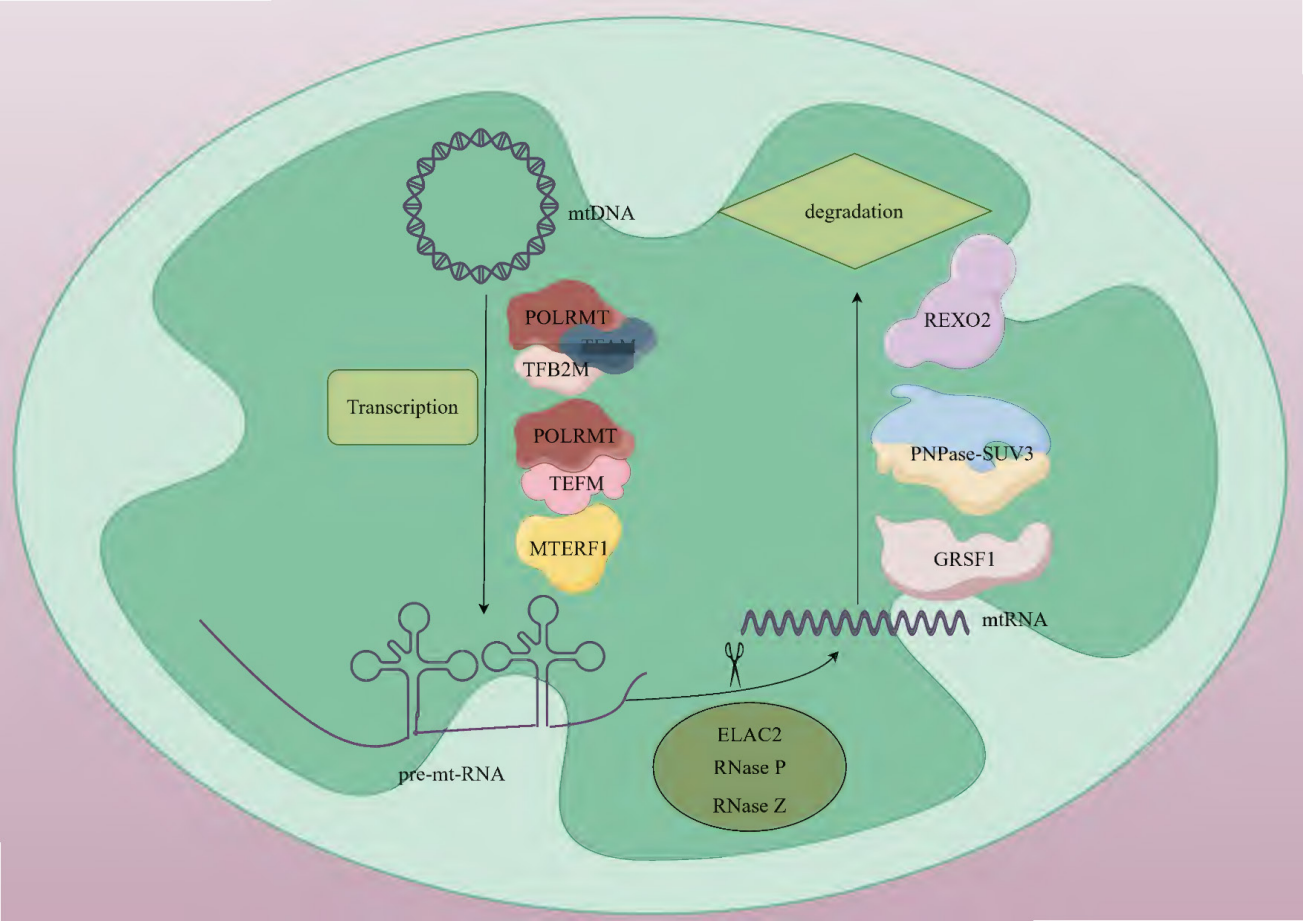
** The basic steps of mtRNA metabolism. Human mtDNA is a circular double-stranded molecule.** Mitochondrial transcription is orchestrated by POLRMT in coordination with its auxiliary factors, including TFAM, TFB2M, TEFM and MTRES1. This process generates three polycistronic transcripts that undergo subsequent processing. During maturation, precursor RNAs are cleaved by ELAC2, RNase P, and RNase Z to form functional mtRNAs. Terminal RNA degradation requires the coordinated action of GRSF1 and PNPase-SUV3. REXO2 ensures proper degradation of mt-dsRNA by eliminating nanoscale RNA fragments produced during SUV3-PNPase-mediated processing. mtDNA; mitochondrial DNA, POLRMT; mitochondrial RNA polymerase, TFAM; mitochondrial transcription factor A, TFB2M; mitochondrial transcription factor B2, TEFM; mitochondrial transcription elongation factor, MTERF1; mitochondrial transcription termination factor 1, pre-mt-RNA; mitochondrial precursor RNA, ELAC2; ElaC domain protein 2, RNase P; ribonuclease P, RNase Z; ribonuclease Z, mtRNA; mitochondrial RNA, GRSF1; G-rich sequence factor 1, SUV3-PNPase; suppressor of variegation 3-polynucleotide phosphorylase, REXO2; RNA exoribonuclease 2.

mtRNA transcription operates through a prokaryote-like mechanism, mediated by mtRNA polymerase (POLRMT) with essential support from mitochondrial transcription factor A (TFAM) and mitochondrial transcription factor B2 (TFB2M) 13, 14. The mitochondrial genome harbors 37 intron-free genes (2 rRNAs, 22 tRNAs, and 13 mRNAs) organized as polycistronic transcription units, which encode 13 critical subunits required for the oxidative phosphorylation (OXPHOS) system 15. Transcription initiation occurs at the light-strand promoter (LSP) and heavy-strand promoters (HSP) within the control region adjacent to the D-loop structure 16. During initiation, TFAM binds high-affinity sites 10-15 base pairs upstream of transcription start sites, inducing DNA bending and interacting with POLRMT to recruit it to promoter regions. TFB2M subsequently joins to form a pre-initiation complex that unwinds promoter DNA, enabling transcription commencement 16, 17. The mitochondrial transcription elongation factor (TEFM) facilitates POLRMT in navigating complex structural regions. The resultant polycistronic RNA undergoes site-specific processing to yield mature mRNAs, tRNAs, and rRNAs. tRNA genes typically flank protein-coding genes, with tRNA maturation triggering release of mRNAs and rRNAs for subsequent maturation steps. Termination is regulated by factors including mitochondrial transcription termination factor 1 (MTERF1) 18 (Fig. 1).

### 2.2 Types and Functions of mtRNA

mtDNA generates polycistronic transcripts through bidirectional transcription, and these long-chain RNAs need to undergo precise processing. mt-mRNA, mt-tRNA, and mt-rRNA are generated through 5'-end cleavage mediated by the TRMT10C-SDR5C1-PRORP complex and 3'-end cleavage catalyzed by ElaC domain protein 2 (ELAC2) 19, 20 (Table. 1). Among them, mt-mRNA encodes 13 protein subunits of the mitochondrial respiratory chain complex. These subunits, through cooperative assembly with nuclear-encoded subunits, form the core functional units of the electron transport chain 21. mt-tRNA is responsible for transporting amino acids to the ribosome. It pairs its anticodon with the codon on mRNA to ensure the correct synthesis of the amino acid sequence. There are 22 types of mt-tRNAs. In most cases, each tRNA corresponds to a specific amino acid, but there are also a few tRNAs that can recognize and bind to several structurally similar amino acids 22. mt-rRNA is a component of the mitochondrial ribosome, including two types, 12S and 16S, which participate in the formation of the small and large subunits, respectively 23. Ribosomes are the sites of protein synthesis. rRNA, together with ribosomal proteins, forms the structural framework of the ribosome and participates in the catalytic reactions during protein synthesis 24. During the transcription of mtDNA, long-chain RNA molecules have a relatively high probability of forming double-stranded RNA (dsRNA) structures through complementary base pairing. After the long-chain RNA molecules formed during mitochondrial transcription form mitochondrial dsRNA (mt-dsRNA) structures, they may be recognized and degraded by nucleases within the mitochondria, or they may participate in some regulatory processes, such as regulating mitochondrial gene expression 25. The formation and degradation of mt-dsRNA are part of the normal operation of mitochondrial functions. The correct processing and degradation of mtRNA are crucial for maintaining mitochondrial functions and cellular energy metabolism 26.

### 2.3 Metabolism of mtRNA

The mtRNA degradation pathway entails the orchestrated activity of various enzymes, which play a crucial role in preserving mtRNA homeostasis by identifying and cleaving RNAs with distinct structures or sequences. This degradation process is primarily executed through the synergistic actions of endonucleases and exonucleases 64. During the initial processing stage, endonucleases Ribonuclease P (RNase P) and Ribonuclease Z (RNase Z) cleave precursor RNAs to generate mature rRNAs, tRNAs, and mRNAs 20, 65. The subsequent terminal degradation is mainly mediated by exonuclease complexes. Exonucleases like polynucleotide phosphorylases (PNPase, PNPT1) and suppressor of variegation 3 homolog (SUV3, Suv3p) are responsible for further degrading these RNA molecules 66. PNPase is a phosphorolytic 3'-5' exoribonuclease that can catalyze the degradation of phosphodiester bonds in RNA and is mainly responsible for the degradation of mitochondrial antisense RNA. In mammals, The localization of PNPase to the mitochondrial inter-membrane space 67 and matrix 68 suggests that it has a dual role in preventing the formation and release of mt-dsRNA into the cytoplasm. PNPase collaborates with SUV3 in the matrix to participate in the degradation of antisense RNA 69. SUV3 is an RNA helicase. In yeast, it is localized in the mitochondrial matrix and forms the core of the degradosome together with Dss1p, catalyzing the unwinding of RNA duplexes to facilitate Dss1p-mediated degradation 70, 71. In mammals, SUV3 and PNPase form the mitochondrial degradosome (mtEXO), which maintains the stability of the mitochondrial genome by unwinding RNA and removing abnormal structures such as R-loops 72. In addition to the core degradation system, other enzymes including MRP1/2, RBP16, LRPPRC, SLIRP, MTPAP, PDE12, and multiple co-factors participate in quality control by regulating RNA stability 73, 74. In recent years, studies have found that the G-rich sequence factor 1 (GRSF1) plays a specific regulatory role in L-strand RNA metabolism. GRSF1 is an RNA-binding protein that localizes to mitochondria and participates in the post-transcriptional processing of mtRNA and the regulation of gene expression. There are two isoforms of GRSF1 in mitochondria. One is localized in mtRNA granules (MRGs), co-localizing with newly synthesized mtRNA and RNase P. The other is localized in the nucleus and cytoplasm and may be involved in mitochondria-to-nucleus communication 75, 76. GRSF1 mainly binds to L-strand RNA, and many L-strand RNAs in mitochondria have the potential to form G-quadruplexes. GRSF1 is a key protein that targets these G-quadruplex RNAs and promotes their degradation 76, 77. GRSF1 acts in concert with the mitochondrial degradosome (the hSuv3-PNPase complex), enhancing the degradation efficiency of G-quadruplex RNAs 76. RNA exoribonuclease 2 (REXO2), an evolutionarily conserved 3'-5' DEDDh family exonuclease, maintains normal degradation of mt-dsRNA by eliminating nanoRNAs (e.g., ncH2 RNA) generated by the mitochondrial SUV3-PNPase complex, thereby preventing their accumulation and interference with PNPase activity. This process is critical for safeguarding against mitochondrial dysfunction 78 (Fig. 1).

### 2.4 mt-dsRNA and mtRNA

mtDNA is characterized by its circular, double-stranded structure, and its expression results in the production of extensive complementary RNA strands, which have a pronounced tendency to form mt-dsRNA 25. In instances where mitochondrial function is compromised, the RNA degradation system, such as the PNPase-SUV3 complex, may become dysfunctional. This dysfunction can lead to the inadequate degradation of single-stranded mtRNA, which subsequently folds into stable mt-dsRNA 78. Consequently, mt-dsRNA represents a distinct subtype of mtRNA, as not all mtRNA molecules exist in a double-stranded form. While mtRNA includes all RNA molecules present within mitochondria, mt-dsRNA specifically refers to those with a double-stranded configuration. Furthermore, mt-dsRNA is more readily recognized by PRRs, which can initiate an inflammatory response.

## 3. Mechanisms of mtRNA Activating Inflammatory Response

### 3.1 Activation of Pattern Recognition Receptors

**Figure 2:**
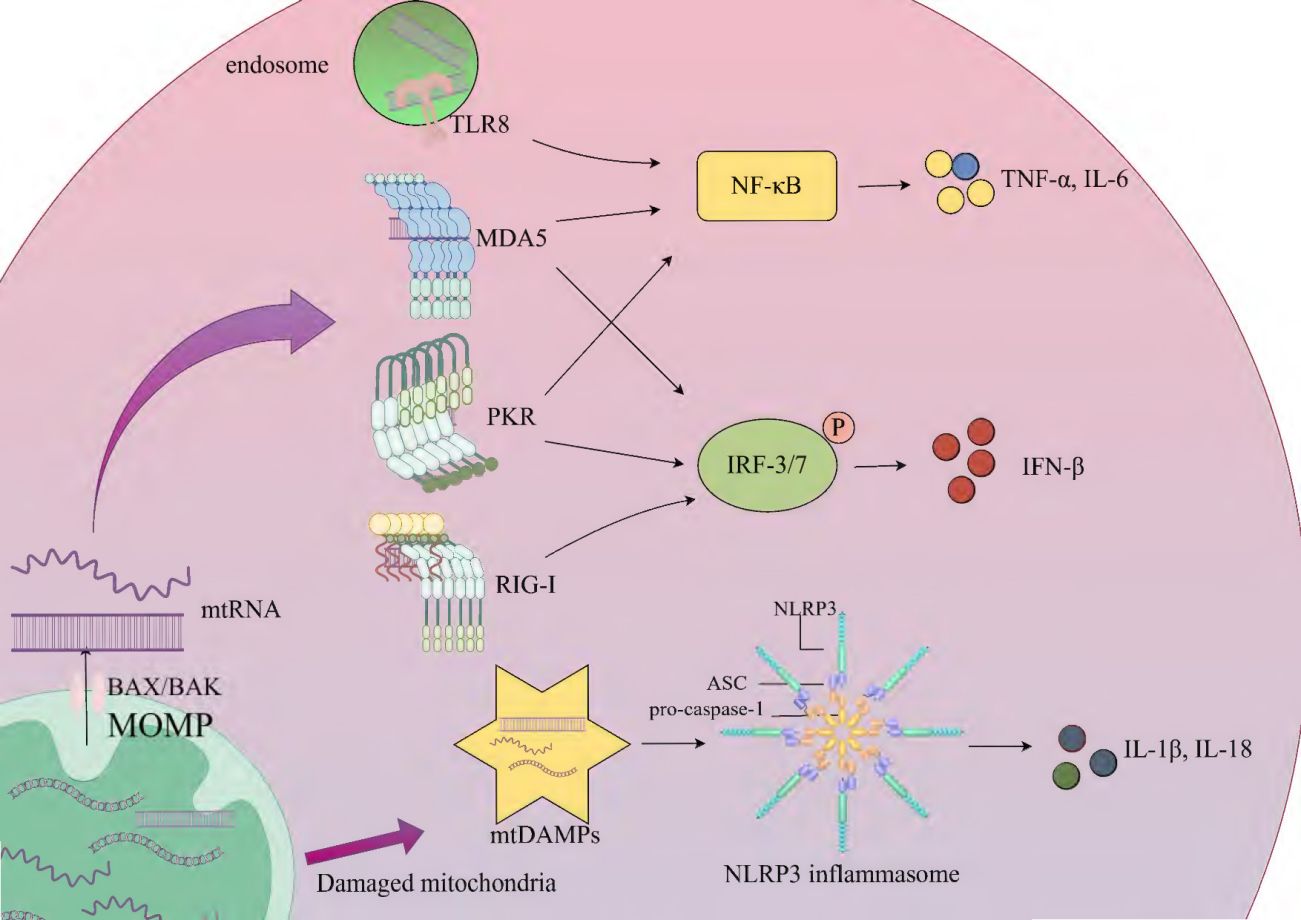
** Activation of the innate immune response by mtRNA. Key steps of the process are presented on the simplified diagram.** Mitochondrial dysfunction triggers stress signals that activate BAX/BAK, which oligomerize and insert into the outer mitochondrial membrane, forming pores that increase MOMP and release mtRNA into the cytoplasm, thereby activating the innate immune response. RIG-I and MDA5 recognize and bind mtRNA, interacting with MAVS to recruit kinase complexes such as TBK1/IKKε, which activate downstream signaling pathways. RIG-I activates IRF3/7 to promote IFN-β expression, while MDA5 activates NF-κB and IRF3/IRF7, inducing the transcription of TNF-α and IL-6, as well as IFN-β expression. mtRNA activates immune cells through the TLR8-mediated pathway, triggering the NF-κB signaling pathway and promoting the secretion of TNF-α and IL-6. This process relies on Unc93B1 to deliver TLR8 to the endosome. mtRNA interacts with PKR, activating NF-κB and IRF3/IRF7-mediated pathways, which upregulate TNF-α, IL-6, and IFN-β. The NLRP3 inflammasome, composed of NLRP3, ASC, and pro-caspase-1, is indirectly activated by mtDAMPs such as mtDNA and mtRNA released into the cytoplasm due to mitochondrial damage. These mtDAMPs bind to the PYD domain of ASC or promote ASC speck formation, ultimately enhancing the expression of IL-1β and IL-18. MOMP; outer mitochondrial membrane permeability, BAX/BAK; BCL-2-associated X protein/BCL-2 antagonist or killer, TLR8; Toll-like receptor 8, MDA5; melanoma differentiation-associated protein 5, PKR; protein kinase R, RIG-I; retinoic acid-inducible gene I, NF-κB; nuclear factor-κB, IRF3/7; interferon regulatory factor 3/7, mtDAMPs; mitochondrial damage-associated molecular patterns, ASC; apoptosis-associated speck-like protein containing a CARD.

Inflammation, a fundamental innate immune response to noxious stimuli such as infection or tissue injury, serves core functions including pathogen clearance, removal of necrotic cells, and initiation of tissue repair 79. This response is initiated through the recognition of two key molecular patterns by pattern recognition receptors: pathogen-associated molecular patterns (PAMPs) and DAMPs. PAMPs represent conserved components of pathogens, such as bacterial lipopolysaccharides and viral double-stranded RNA, while DAMPs encompass endogenous molecules released by host cells during injury or stress, including high mobility group box-1 (HMGB1), adenosine triphosphate (ATP), and mitochondrial components (mtDNA and mtRNA) 80. Upon cellular damage, mtRNA (especially mt-dsRNA) may leak into the cytosol or extracellular space, acting as DAMPs recognized by PRRs. However, certain PRRs (e.g., RIG-I-like receptors), which primarily evolved to detect exogenous PAMPs (e.g., viral RNA), exhibit cross-reactivity toward endogenous mtRNA, potentially leading to aberrant activation of analogous inflammatory pathways 80, 81. Under physiological conditions, the level of mtRNA in mitochondria is regulated by mtRNA degradation complexes such as PNPase and SUV3. When mitochondrial function is impaired (such as metabolic stress in the tumor microenvironment), stress signals activate the pro-apoptotic proteins BAX (BCL-2-associated X protein) and BAK (BCL-2 antagonist or killer). They oligomerize and insert into the outer mitochondrial membrane to form pores, leading to an increase in outer mitochondrial membrane permeability (MOMP). This process results in the release of mtRNA and mtDNA into the cytoplasm 9. Double-strand breaks in mtDNA can lead to its linearization and degradation. Meanwhile, mtRNA polymerase generates short-chain mtRNA transcripts at the break sites. These transcripts carry 5'-triphosphate (5'PPP) ends and are partially fold into short-chain mt-dsRNA, which are released into the cytoplasm through BAK/BAX pores. The mtRNA that enters the cytoplasm activates RIG-I-like receptors (RLRs), such as RIG-I and MDA5 82. Nucleic acid binding induces conformational changes in RIG-I and MDA5, exposing their N-terminal caspase recruitment domains (CARDs). Subsequently, the CARDs polymerize and interact with the CARD domain of mitochondrial antiviral-signaling protein (MAVS), ultimately triggering a cascade of antiviral signals such as type I interferon 9, 83. MAVS is located on the outer mitochondrial membrane and serves as a key node in innate immune defense. It can activate downstream signaling pathways by recruiting kinase complexes (such as TBK1/IKKε), including the nuclear factor-κB (NF-κB) and interferon regulatory factor 3/7 (IRF3/7) signaling pathways. The activated NF-κB and IRF3/IRF7 can promote the transcription of related inflammatory factors (such as TNF-α, IL-6) and the expression of type I interferon (such as IFN-β), respectively, thereby triggering an inflammatory response 84. In addition, fumarase (FH) catalyzes the conversion of fumarate to malate in the mitochondria and cytoplasm of macrophages. When macrophages are treated with the FH inhibitor FHIN1 or a macrophage model with Fh1 gene knockout is constructed by gene editing, intracellular fumarate accumulates, and there is an increase in mtRNA release. In experiments where macrophages stimulated with lipopolysaccharide (LPS) were treated with FHIN1, researchers found that the content of mtRNA in cytoplasmic extracts increased significantly. In tamoxifen-induced Fh1-knockout bone-marrow-derived macrophages (BMDMs), an increase in mtRNA release was also observed. This indicates that FH inhibition is an important cause of increased mtRNA release, and the mtRNA released into the cytoplasm can activate the immune response 85. Toll-like receptors (TLRs) mediate the recognition of pathogen- and host-derived "danger-associated molecular patterns" (P-/DAMPs), triggering a severe inflammatory response. Human TLR8 can specifically recognize the UR/URR motifs in bacterial and mtRNA. mtRNA activates immune cells through the human TLR8-mediated pathway, inducing the MyD88 (myeloid differentiation primary response 88) adaptor-mediated NF-κB signaling pathway, thus promoting the secretion of pro-inflammatory factors (such as TNF-α, IL-6). This process depends on the endosomal transporter Unc93B1 to deliver TLR8 to the endosome and is independent of TLR7 86. Studies have demonstrated that mitochondrial stress conditions, particularly those associated with mitochondrial dysfunction and aging, significantly enhance cytosolic translocation of mt-dsRNAs. These translocated mt-dsRNAs interact with PKR, inducing its dimerization and subsequent autophosphorylation. The phosphorylated PKR (pPKR) functions as an active kinase that phosphorylates eIF2α to suppress global protein synthesis while simultaneously activating NF-κB-mediated pathways. This dual mechanism upregulates pro-inflammatory cytokines (e.g., IL-8, MMP1/13, IFN-β) and enhances expression of interferon-stimulated genes (ISGs) 87, 88. The NLR family pyrin domain containing 3 (NLRP3) inflammasome is a multi-protein complex composed of NLRP3, apoptosis-associated speck-like protein containing a CARD (ASC), and pro-caspase-1. Mitochondrial damage leads to the release of mitochondrial damage-associated molecular patterns (mtDAMPs), including mtDNA and potentially mtRNA. These mtDAMPs can indirectly activate the NLRP3 inflammasome by binding to the PYD domain of ASC or promoting the formation of ASC specks. After activation, ASC recruits pro-caspase-1 through its CARD domain and promotes its self-cleavage into the active form caspase-1. The latter then cleaves the pro-inflammatory cytokines IL-1β and IL-18 into their active forms 89, 90 (Fig. 2).

### 3.2 Transcellular Inflammatory Regulatory Effects

**Figure 3:**
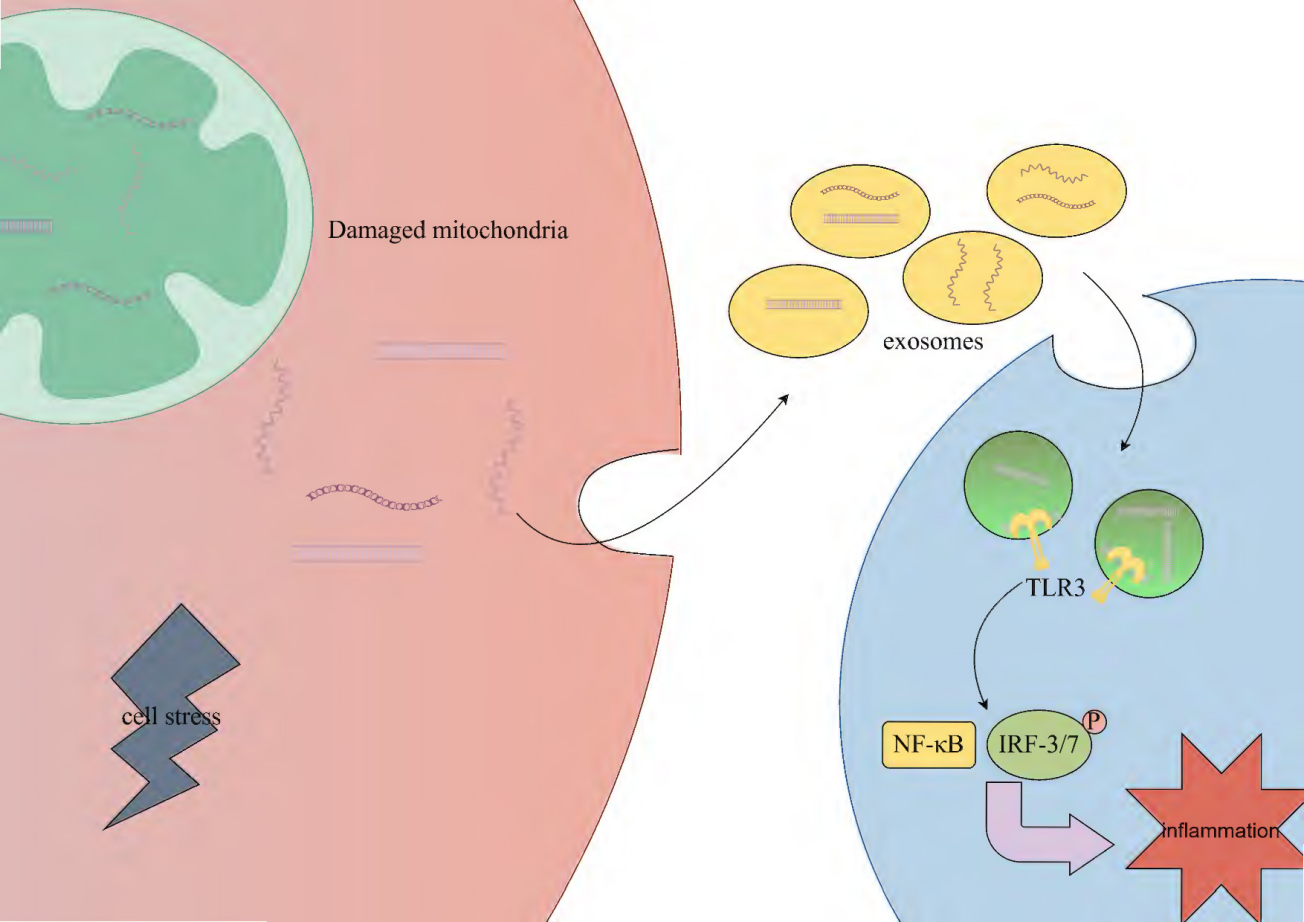
** Stress-induced mtRNA release triggers inflammation in neighboring cells.** Under cellular stress or injury conditions, mt-dsRNA may be encapsulated into exosomes through membrane fusion-mediated extracellular release. These vesicles are subsequently internalized by neighboring cells via endocytic mechanisms. Intracellular recognition of mt-dsRNA by TLR3 triggers the activation of both IRF3/7 and NF-κB signaling pathways, ultimately driving inflammatory responses. TLR3; toll-like receptor 3, NF-κB; nuclear factor-κB, IRF3/7; interferon regulatory factor 3/7.

Circulating extracellular vesicles (EVs), including exosomes, microvesicles, and apoptotic bodies, can carry various molecules (such as DNA, RNA) and transmit information between cells 91, 92. Under conditions of cell stress or injury, mt-dsRNA may be packaged into exosomes. Exosomes are released extracellularly through fusion with the cell membrane and can be taken up by other cells via endocytosis or other mechanisms, thus enabling intercellular communication and information transfer. However, the specific mechanisms remain unclear 91, 93. The mt-dsRNA released into other cells may activate TLR3, thereby mediating the IRF3/7 and NF-κB signaling pathways, which subsequently promote the transcription of relevant inflammatory cytokines and the expression of type I interferons 94 (Fig. 3).

### 3.3 Nuclear Regulatory Mechanisms of mtRNA

In the field of diabetes research, under conditions simulating diabetes (high glucose and TNF-α treatment), the association level between mtRNA and chromatin in human umbilical vein endothelial cells (HUVECs) increases. In particular, the association level of SncmtRNA (a mitochondrially encoded long non-coding RNA) significantly increases under stress conditions. Through the combined analysis of single-cell RNA sequencing (scRNA-seq) and single-nucleus RNA sequencing (snRNA-seq), researchers found that the knockdown of SncmtRNA (Snc-KD) significantly inhibited the expression of intercellular adhesion molecule 1 (ICAM1) and vascular cell adhesion molecule 1 (VCAM1). These genes are induced under stress conditions and are closely related to the inflammatory response. Results indicate that mtRNA can act as a retrograde signaling molecule to regulate gene expression through its interaction with nuclear chromatin. In addition, SncmtRNA plays an important role in regulating the expression of genes related to inflammation and immune responses 95.

## 4. Mechanisms of mtRNA in Various Inflammatory-related Diseases

### 4.1 Autoimmune Disease

SLE is an autoimmune-mediated connective tissue disorder, characterized by the production of multiple autoantibodies and multisystem damage. The mechanisms by which mt-dsRNA activates the inflammatory response suggest that damage-associated molecular patterns, such as mt-dsRNA released due to mitochondrial dysfunction, may engage the innate immune response via pattern recognition receptors, including RIG-I and MDA5, thereby contributing to the pathogenesis of SLE. mt-dsRNA has been identified as a novel mitochondrial autoantigen target in SLE 96. In individuals with SLE, autoantibodies against mtRNA (AmtRNA) are present, with research indicating that the serum levels of IgG and IgM subtypes of AmtRNA are significantly elevated compared to healthy controls. The presence of AmtRNA-IgG correlates with anti-mtDNA-IgG, as well as IgG and IgM against anti-β2-glycoprotein I, with even higher levels observed in patients who are positive for anti-double-stranded genomic DNA antibodies. These findings suggest that mt-dsRNA functions as an autoantigen, capable of activating the immune system, inducing the production of autoantibodies, and contributing to the immunopathological processes underlying SLE 96. Mitochondria-derived mt-dsRNA from erythrocytes plays an important role in SLE. When monocytes in SLE patients phagocytose erythrocytes containing mitochondria (Mito+RBCs), the mt-dsRNA derived from Mito+RBCs can trigger monocytes to release their own mtDNA fragments. These fragments bind to the NLRP3 inflammasome, promoting the production of IL-1β. At the same time, mt-dsRNA also acts in synergy with type I interferon. The MxA protein induced by type I interferon is involved in the unconventional secretion pathway of IL-1β, affecting the inflammatory response 97.

Rheumatoid arthritis (RA) is a complex chronic autoimmune disease. Its main pathological features include progressive joint deformity, synovial inflammation, and cartilage and bone destruction. Its pathogenesis is closely related to mitochondrial dysfunction. Due to the inflammatory-activating effect of mt-dsRNA, when mitochondria are damaged, mt-dsRNA leaks into the cytoplasm, activating the innate immunity and thus releasing inflammatory factors. This process is particularly significant in RA synovial cells, exacerbating joint inflammation 98. Meanwhile, the release of mt-dsRNA is accompanied by the accumulation of mitochondrial reactive oxygen species (mtROS), which activates the NLRP3 inflammasome and promotes the maturation and secretion of IL-1β and IL-18. Clinical data show that the level of IL-1β in the synovial fluid of RA patients is positively correlated with mtROS 99.

Sjögren's syndrome (SS) is an autoimmune disease primarily characterized by damage to exocrine glands such as salivary glands and lacrimal glands, presenting symptoms like xerostomia and xerophthalmia 88, 100. Its pathogenesis is closely related to innate immune abnormalities and activation of IFN signaling. The levels of mt-dsRNAs are elevated in the saliva, tears, and salivary glands of SS patients. These mt-dsRNAs can be recognized by pattern recognition receptors such as PKR, MDA5, and TLR3, activating the innate immune response, which in turn activates the Janus Kinase-1 (JAK1) / signal transducer and activator of transcription (STAT) signaling pathway. As a key kinase in this pathway, JAK1 mediates the downstream transmission of IFN signals, promoting the expression of interferon-stimulated genes (ISGs) and forming an inflammatory amplification loop, thereby exacerbating glandular inflammation and dysfunction 88, 100.

### 4.2 Degenerative Disease

Osteoarthritis (OA) is a common degenerative joint disease that mainly affects the synovial joints of adults and is the most common type of arthritis. It often occurs in the knee, hip, and hand joints 101. The over-activation of PKR is associated with inflammatory degenerative diseases including OA, but the dsRNA molecules that activate it remain largely unclear. Researchers have found that under conditions that induce osteoarthritis, the expression of mt-dsRNA in chondrocytes and its outflow into the cytoplasm are accelerated, leading to innate immune activation 11.

Huntington's disease (HD) is an inherited neurodegenerative disease caused by a mutation in the HTT gene, which encodes the huntingtin protein (Htt). Research has found that in patients with HD and mouse models, the up-regulation of mt-dsRNA expression is related to disease progression. The immune response induced by mt-dsRNA may be involved in the increased vulnerability of the neurons most severely affected in HD patients (striatal projection neurons). In addition, the accumulation of mtRNA in senescent cells promotes neuro-inflammation through the RIG-I/MDA5-MAVS 102, 103.

### 4.3 Liver Disease

Alcoholic liver disease (ALD) is an inflammatory liver disease caused by long-term and excessive alcohol consumption, which is mediated by multiple inflammatory responses. Research shows that ethanol stress prompts hepatocytes to produce mt-dsRNA. These mt-dsRNA are transmitted to Kupffer cells via exosomes, activating TLR3 in them 104. Studies have found that treating wild-type Kupffer cells with exosomes directly treated with ethanol (EtOH-Exo) can significantly increase the expression of IL-1β, but this phenomenon does not occur in TLR3-knockout Kupffer cells. This indicates that mt-dsRNA activates TLR3 through exosomes to initiate the immune response. The activated TLR3 makes Kupffer cells express IL-1β, which in turn stimulates γδ T cells to produce IL-17A, participating in the inflammatory response of ALD 104.

Non-alcoholic fatty liver disease (NAFLD) is a type of metabolic stress-induced liver injury disease closely associated with insulin resistance and genetic susceptibility 105. As a key mitochondrial damage-associated molecular pattern, mt-dsRNA is released into the cytoplasm under certain pathophysiological conditions, triggering innate immune responses. In the alcoholic liver disease model, it can induce the pro-inflammatory activity of Kupffer cells. Studies have shown that a high-fat diet (HFD) can induce the release of mt-dsRNA in wild-type (WT) mice. As a critical DAMP, mt-dsRNA triggers a series of innate immune responses. Specifically, the release of mt-dsRNA activates TLR3, MDA5, and phosphorylated interferon regulatory factor 3 (p-IRF3). This further leads to the upregulated mRNA expression of inflammatory biomarkers such as il-1β and il-6 in the liver, thereby causing hepatic inflammation 106. In addition, studies have noted that in the alcoholic liver disease model, after being encapsulated by exosomes, mt-dsRNA is transferred from hepatocytes to Kupffer cells in a TLR3-dependent manner, triggering their pro-inflammatory activity. This also supports the role of mt-dsRNA in triggering innate immune responses 106.

### 4.4 Kidney Disease

Renal tubular atrophy is a key feature of chronic kidney disease (CKD). Studies have found that downregulation of PNPT1 in renal tubular cells leads to leakage of mt-dsRNA into the cytoplasm, activation of PKR, subsequent phosphorylation of eIF2α, and termination of protein translation and apoptosis. Downregulation of PNPT1 and activation of the mt-dsRNA-PKR-eIF2α axis have been observed in human kidney disease patients such as acute tubular necrosis (ATN), diabetic nephropathy (DN), and lupus nephritis (LN), as well as in mouse models of ischemia-reperfusion injury (IRI) and unilateral ureteral obstruction (UUO). Overexpression of PNPT1 or inhibition of PKR activity significantly alleviates renal tubular injury, while renal tubular-specific PNPT1 knockout mice exhibit Fanconi syndrome-like phenotypes and severe mitochondrial damage 107.

DN is a common microvascular complication of diabetes mellitus, characterized by hyperglycemia, persistent increase in urinary albumin excretion, and progressive decline in glomerular filtration rate (GFR). In both type 1 (STZ-induced) and type 2 (db/db) DN mouse models, the expression of mt-dsRNA in tubular cells is significantly increased, with abnormal release from mitochondria to the cytoplasm. After being released into the cytoplasm, mt-dsRNA activates the double-stranded RNA sensor PKR, inducing its phosphorylation (p-PKR), which in turn promotes the phosphorylation of downstream eIF2α (p-eIF2α), ultimately leading to tubular cell apoptosis. In DN mice and HK-2 cells treated with high glucose (HG), elevated mt-dsRNA is accompanied by increased expression of pro-apoptotic proteins (BAX, Cleaved caspase-3) and decreased expression of anti-apoptotic protein (BCL-2); while inhibiting mt-dsRNA release or blocking PKR activation can reduce apoptosis 108.

### 4.5 Infectious Disease

Viral infections can induce abnormal accumulation of mt-dsRNA in mammalian cells. When these mt-dsRNA are released into the cytoplasm through mitochondrial membrane channels or the mitophagy pathway, they can activate cytoplasmic pattern recognition receptors (such as MDA5/RIG-I), triggering the host antiviral immune response (such as the type I interferon pathway) 109.

### 4.6 Cancer

The mutational signature of the antiviral DNA deaminase APOBEC has been identified in over 70% of cancers and may represent the predominant component of somatic variations in numerous individual tumors and tumor types 110, 111. Research has demonstrated that the cytoplasmic leakage of mt-dsRNA is perceived as foreign nucleic acid, thereby activating the interferon response, including the upregulation of APOBEC3A via the RIG-I/MAVS/STAT2-dependent pathway, while concurrently inducing nuclear DNA damage 112. In B-cell precursor acute lymphoblastic leukemia (B-ALL), studies have indicated that mt-dsRNA serves as a pivotal trigger for the transformation of mesenchymal stromal cells (MSCs) into cancer-associated fibroblasts (CAFs). This transformation is characterized by a robust interferon pathway response, which includes the upregulation of interferon pathway-related genes such as MX1, MX2, and OAS1-3, alongside the secretion of IFN-β. The majority of these genes are implicated in RNA sensing or inhibition processes 113.

### 4.7 Cardiovascular Disease

Atherosclerosis (AS) is a pathological change in the early stage of cardiovascular diseases, with a high incidence in middle-aged and elderly people. Endothelial cell injury is the initiating factor in the early stage of AS, which can lead to plaque instability and rupture, accelerating the progression of the disease. Endothelial cell injury can be caused by multiple factors, including hyperlipidemia, oxidative stress, and mechanical stress. The release of mtRNA into the cytoplasm, through the activation of PRRs and the NLRP3 inflammasome activation pathway, exacerbates vascular inflammation, disrupts the endothelial barrier function, increases vascular permeability, promotes monocyte infiltration, and lipid deposition. Abnormal mtRNA may promote the development of AS by generating mitochondrial ROS, oxidizing LDL (ox-LDL), and promoting the formation of foam cells 114, 115.

### 4.8 Respiratory Disease

Asthma is a widely prevalent chronic respiratory disorder that is defined by airway inflammation, heightened bronchial responsiveness, and intermittent airflow obstruction. One of the inflammatory response mechanisms through which mt-dsRNA exerts its effects involves PRRs, particularly TLR3 and the RLRs. Upon detection of mt-dsRNA, these receptors initiate downstream signaling cascades, resulting in the production of pro-inflammatory cytokines, including IFNs, IL-6, and TNF-α 116. Experimental data indicate a positive correlation between cytoplasmic mt-dsRNA levels and the expression levels of IL-6, IL-5, and IL-13, with a notable elevation of Th2 cytokines in the lungs. Research has demonstrated that epithelial ETS2 is upregulated in asthma and that ETS2 in epithelial cells enhances IL-6, IL-5, and IL-13 levels through ANT2-mediated regulation of mt-dsRNA levels in the cytosol 116.

## 5. Metabolic Regulation of mtRNA and Inflammation

During the inflammatory response, PNPase plays a multi-level immunoregulatory role by regulating mtRNA metabolism, maintaining the stability of the mitochondrial genome, and the homeostasis of energy metabolism, and participating in the regulation of signaling pathways 69. PNPase and SUV3 form a complex in the mitochondrial matrix, where they synergistically degrade single-stranded mtRNA and unwind dsRNA , effectively preventing the abnormal accumulation of dsRNA. When the function of this complex is impaired, mt-dsRNA accumulates within mitochondria 83. In addition, The loss of PNPase leads to an accumulation of mt-dsRNA that exceeds the normal retention capacity of mitochondria, which is then released through voltage-dependent anion channels 1/2 (VDAC1/2) and BAK/BAX channels, activating the cytoplasmic pattern recognition receptor MDA5 and triggering excessive type I interferon responses and inflammatory cascades 78, 118, 119. SUV3 deficiency leads to the accumulation of dsRNA into the mitochondrial matrix, which triggers MDA5-mediated antiviral signaling pathways and type I interferon responses. However, unlike PNPase, SUV3 deficiency does not directly result in the release of dsRNA into the cytoplasm 68, 118. This functional difference stems from their division of labor in the complex: SUV3 disrupts RNA secondary structures through its helicase activity, while PNPase degrades linearized RNA with its 3'-5' exonuclease activity 120, 121.

## 6. mtRNA as an Inflammatory Biomarker and Therapeutic Target

Under various disease conditions, changes in mtRNA are closely associated with the inflammatory response. Its stability in peripheral body fluids (such as serum) indicates its potential as an inflammatory biomarker. In the autoimmune disease SS, stimulation with polyinosinic-polycytidylic acid (poly I:C) increases the levels of total mt-dsRNA and cytosolic mt-dsRNA in salivary gland acinar cells, followed by an increase in PKR phosphorylation and enhanced induction of ISGs. Downregulating mt-dsRNAs with 2-CO-methyladenosine (2-CM) attenuates the IFN signaling signature 88.

Studies have confirmed that mt-dsRNA is closely associated with Sjögren's syndrome in terms of expression levels, disease activity, and differential diagnosis. In terms of expression levels, there are significant differences in mtRNA levels in saliva and plasma between SS patients and healthy individuals. For example, plasma ND1 levels decrease and ND4 levels increase in Sjögren's syndrome patients; saliva ND1 levels increase, while ND5 and others decrease in SS patients. Regarding disease activity, saliva mtRNA scores are positively correlated with objective disease activity indicators (such as ESSDAI and ClinESSDAI) and Raynaud phenomenon, and some plasma mtRNAs are positively correlated with patients' global assessment. In terms of differential diagnosis, the ability of mtRNA composite scores to distinguish patients from healthy individuals is better than that of ISG scores, and the plasma mtRNA scores of patients are significantly higher than those of patients with rheumatoid arthritis and systemic lupus erythematosus, which is conducive to differentiation. This indicates that mtRNA may be a potential biomarker for disease monitoring and stratification in SS 100.

Besides autoimmune diseases, in degenerative diseases like OA, changes in mtRNA are also closely related to the inflammatory response. The expression of mt-dsRNA is significantly increased in the damaged cartilage of OA patients, synovial fluid, and the cartilage of surgically induced OA mice. The level of mt-dsRNA in the cartilage of patients is positively correlated with the senescence-associated secretory phenotype (SASP) and the expression of ISGs, and it increases with the aggravation of cartilage damage 11. Many studies have shown that activating autophagy can clear mt-dsRNA in the cytoplasm, thereby rescuing the phenotype of mitochondrial dysfunction and alleviating OA-related changes in articular chondrocytes 11.

In liver diseases, research on ALD has also revealed the association between mt-RNA and the inflammatory response. Immunostaining shows that in ethanol-treated mice and hepatocytes of healthy individuals, mt-dsRNA co-localizes with mitochondria, indicating that the accumulation of mt-dsRNA is related to the inflammatory state of hepatocytes under alcohol stimulation and can serve as a potential inflammatory biomarker 104. From a therapeutic perspective, studies have found that increasing the expression level of PNPase or enhancing its activity can effectively reduce the accumulation of mt-dsRNA. Meanwhile, experiments have demonstrated that when Kupffer cells lack TLR3, the migration of γδ T cells co-cultured with them and the expression of IL-17A are abnormal. This directly indicates that TLR3 is a crucial link for Kupffer cells to receive mt-dsRNA signals and transmit them to γδ T cells, enabling the production of IL-17A and cell migration. It further proves the indispensability of TLR3 in the mt-dsRNA-mediated inflammatory response, suggesting that developing antagonists targeting TLR3 can inhibit the inflame-matory response and providing a potential therapeutic target for ALD 104.

Existing studies have confirmed that cytoplasmic leakage of mt-dsRNA drives the SASP through the RIG-I/MDA5-MAVS pathway, triggering inflame-matory responses, and this process depends on mitochondrial membrane permeability changes mediated by BAX/BAK. BAX/BAK, RIG-I/MDA5, and MAVS may serve as potential targets for inhibiting senescence-related inflammation, provi-ding new insights for intervening in age-related diseases such as tissue fibrosis 122.

## 7. Current Perspectives and Future Directions

As one of the key regulators of mitochondrial function, the interaction mechanism between mtRNA and the inflammatory response has become a research frontier in the field of immunometabolism. With the role of mtRNA in inflammation regulation being gradually revealed, it is of increasing importance to further explore the specific mechanisms of mtRNA in different inflammation-related diseases, especially its dynamic changes during disease occurrence, development, and outcome, as well as its regulatory mechanisms. Verifying the feasibility and accuracy of using the levels of mtRNA in blood or other body fluids as early diagnostic markers for inflammation-related diseases can provide a reliable basis for clinical diagnosis. For example, in certain autoimmune and infectious diseases, the levels of mtRNA are significantly elevated and are positively correlated with disease activity.

Regarding intervention strategies targeting mtRNA, current research focuses on regulating its metabolism and inflammation-activation pathways. For instance, enhancing the activity of PNPase may promote mtRNA degradation, thereby suppressing mitochondrion-derived inflammation 80. Addi-tionally, by activating mitophagy (such as using inducers like urolithin A) to remove dysfunctional mitochondria, abnormal mtRNA release can be indirectly reduced (as seen in studies related to NLRP3 inflammasome activation). However, the specificity and safety of these therapies still need further evaluation. For example, the role of mitophagy is context-specific: in chronic or mild stress, it exerts a protective effect by clearing damaged mitochondria and inhibiting inflammation (such as limiting the overactivation of the NLRP3 inflammasome); however, in acute and severe infectious inflammation, its excessive activation may suppress the immune response, hinder pathogen clearance, and produce harmful effects 123. During systemic administration, PNPase enhancers may non-specifically affect RNA in other tissues, potentially causing side effects and leading to off-target effects.

Mitochondria play a crucial role in aging-related inflammation. Mitochondria regulate the secretion of aging-related cytokines and chemokines through multiple pathways. The release of signaling components such as mtDNA, mtRNA, N-formylated peptides, and ROS can activate the inflammatory response. Future research can further clarify the specific mechanisms of mt-dsRNA release and its interactions with other aging-related signaling pathways 84. For example, the POLRMT inhibitor IMT1 can reduce mtRNA synthesis, thereby decreasing the cytoplasmic release of mt-dsRNA and effectively alleviating inflammation 124.

## Figures and Tables

**Table 1:** Human mt-RNA Gene Names and Functions.

RNA Type	Gene Name	Function	References
mt-rRNA	MT-RNR1(12S)	Structural component of the mitochondrial ribosome small subunit (28S), involved in codon-anticodon pairing.	27
	MT-RNR2(16S)	Forms the peptidyl transferase center in the large subunit (39S), catalyzing peptide bond formation.	27
mt-mRNA	MT-ND1	Encodes NADH dehydrogenase (Complex I) subunit ND1, involved in electron transport initiation.	28
	MT-ND2	Encodes Complex I subunit ND2, participates in the proton transport process across the mitochondrial inner membrane, working in concert with ND1 to maintain the structural integrity and functional activity of Complex I.	29
	MT-ND3	Encodes Complex I subunit ND3, Ensures the proper assembly of Complex I and maintains the homeostasis of the mitochondrial respiratory chain.	30
	MT-ND4	Encodes Complex I subunit ND4, participates in proton translocation and electron transfer, playing a critical role in the assembly of the membrane arm of Complex I.	31
	MT-ND4L	Encodes Complex I subunit ND4L (smallest transmembrane subunit), participates in proton translocation in Complex I, assisting in maintaining the transmembrane proton gradient.	32
	MT-ND5	Encodes Complex I subunit ND5 (critical for proton pumping), maintains the activity of NADH:ubiquinone oxidoreductase and ensures the proper assembly and stability of the membrane arm of Complex I.	31
	MT-ND6	Encodes Complex I subunit ND6 (encoded on the light strand), collaborates with ND4L and ND5 to maintain the structural stability of Complex I.	33
	MT-CYTB	Encodes cytochrome b (Complex III) (essential for ubiquinol-cytochrome c oxidoreductase activity), participates in the transfer of electrons from coenzyme Q to cytochrome c.	34
	MT-CO1	Encodes cytochrome c oxidase (Complex IV) subunit CO1 (largest catalytic subunit), facilitates the transfer of electrons from cytochrome c to molecular oxygen.	35, 36
	MT-CO2	Encodes Complex IV subunit CO2, participates in electron transfer and stabilizes the structural integrity of Complex IV.	35, 37
	MT-CO3	Encodes Complex IV subunit CO3, assists in proton translocation across the membrane, sustaining the activity of Complex IV.	35, 38
	MT-ATP6	Encodes ATP synthase (Complex V) subunit ATP6 (forms the transmembrane proton channel), participates in the formation of proton channels within the mitochondrial inner membrane, driving ATP synthesis.	39
	MT-ATP8	Encodes ATP synthase (Complex V) subunit ATP8 (supports proton pumping), forms a bicistronic transcript with ATP6, facilitating the assembly and functionality of the ATP synthase complex.	40
mt-tRNA	tRNA-Ala (MT-TA)	Identifying codons GCA, GCC, GCG, GCU, transports alanine.	41
	tRNA-Arg (MT-TR)	Identifying codons CGA, CGC, CGG, CGU, transports arginine.	42, 43
	tRNA-Asn (MT-TN)	Identifying codons AAC, AAU, transports asparagine.	44
	tRNA-Asp (MT-TD)	Identifying codons GAC, GAU, transports aspartic acid.	45
	tRNA-Cys (MT-TC)	Identifying codons UGC, UGU, transports cysteine.	46
	tRNA-Gln (MT-TQ)	Identifying codons CAA, CAG, transports glutamine.	47
	tRNA-Glu (MT-TE)	Identifying codons GAA, GAG, transports glutamic acid.	48
	tRNA-Gly (MT-TG)	Identifying codons GGA, GGC, GGG, GGU, transports glycine.	49
	tRNA-His (MT-TH)	Identifying codons CAC, CAU, transports histidine.	50
	tRNA-Ile (MT-TI)	Identifying codons AUA, AUC, AUU, transports isoleucine.	51
	tRNA-Leu(UUR) (MT-TL1)	Identifying codons UUA, UUG, transports leucine.	52
	tRNA-Leu(CUN) (MT-TL2)	Identifying codons CUA, CUC, CUG, CUU, transports leucine.	53
	tRNA-Lys (MT-TK)	Identifying codons AAA, AAG, transports lysine.	54
	tRNA-Met (MT-TM)	Identifying codons AUA, AUG, transports methionine.	55
	tRNA-Phe (MT-TF)	Identifying codons UUC, UUU, transports phenylalanine.	56
	tRNA-Pro (MT-TP)	Identifying codons CCA, CCC, CCG, CCU, transports proline.	57
	tRNA-Ser(UCN) (MT-TS1)	Identifying codons UCA, UCC, UCG, UCU, transports serine.	58
	tRNA-Ser(AGY) (MT-TS2)	Identifying codons AGC, AGU, transports serine.	59
	tRNA-Thr (MT-TT)	Identifying codons ACA, ACC, ACG, ACU, transports threonine.	60
	tRNA-Trp (MT-TW)	Identifying codons UGG, UGA, transports tryptophan.	61
	tRNA-Tyr (MT-TY)	Identifying codons UAC, UAU, transports tyrosine.	62
	tRNA-Val (MT-TV)	Identifying codons GUA, GUC, GUG, GUU, transports valine.	63

**Table 2:** Mechanisms of mtRNA in Various Inflammatory-related Diseases

Disease category​	Disease name	Possible mechanism	Potential therapeutic strategies	References
Autoimmune disease	Systemic lupus erythematosus	1.mt-dsRNA, functioning as an autoantigen, induces the production of AmtRNA-IgG/IgM by activating cytoplasmic PRRs; 2.After being phagocytosed by monocytes, erythrocyte-derived mt-dsRNA triggers monocytes to release mtDNA fragments. These fragments bind to the NLRP3 inflammasome to promote the production of IL-1β, and synergize with type I interferons to exacerbate inflammation.	1.Enhance the activity of the PNPase/SUV3 complex 2.Block the activation of the RIG-I/MDA5-MAVS signaling axis or the NLRP3 inflammasome.	96, 97
	Rheumatoid arthritis	1.Mitochondrial damage leads to the leakage of mt-dsRNA into the cytoplasm, activates innate immune pathways, and induces the release of proinflammatory cytokines; 2.Along with the accumulation of mtROS, it further activates the NLRP3 inflammasome, exacerbating synovial inflammation and joint damage.	1.Clear cytoplasmic mt-dsRNA through autophagy regulation 2.Inhibit the activity of the NLRP3 inflammasome 3.Reduce mtROS production to decrease mt-dsRNA release	98, 99
	Sjögren's syndrome	mt-dsRNAs can be recognized by pattern recognition receptors such as PKR, MDA5, and TLR3, activating the innate immune response	1.JAK1 inhibitors suppress the synthesis or release of mtRNA 2.Ach activates M3 receptors to inhibit mtRNA release and ISG expression	88, 100
Degenerative disease	Osteoarthritis	In chondrocytes, abnormal accumulation of mt-dsRNA and its efflux into the cytoplasm activate PKR, induce the production of proinflammatory cytokines and cell apoptosis, and accelerate cartilage degradation.	1.Inhibit PKR activity via PKR antagonists 2.Enhance the mtRNA degradation system	11, 101
	Huntington disease	Upregulated expression of mt-dsRNA activates neuroinflammation via the RIG-I/MDA5-MAVS signaling pathway, exacerbates damage to striatal projection neurons.	1.Block the interaction between RIG-I/MDA5 and MAVS 2.Enhance the function of mitochondrial degradation systems to reduce mt-dsRNA accumulation	102, 103
Liver disease​	Alcoholic liver disease	Ethanol stress induces hepatocytes to produce mt-dsRNA, which is delivered to Kupffer cells via exosomes, activates TLR3, induces IL-1β secretion, thereby stimulating γδ T cells to produce IL-17A, and exacerbates hepatic inflammation.	1.TLR3 antagonists inhibit TLR3 activity 2.Block exosome-mediated intercellular transfer of mt-dsRNA	104
	Non-alcoholic fatty liver disease	mt-dsRNA released due to mitochondrial dysfunction upregulates the expression of inflammatory factors by activating the TLR3, MDA5, and p-IRF3 pathways, and is transferred via exosomes to amplify inflammation.	1.Enhance mitophagy to clear damaged mitochondria and abnormal mtRNA 2.Regulate lipid metabolism to improve mitochondrial function	106, 117
Kidney disease	Chronic kidney disease	Downregulation of PNPT1 leads to the leakage of mt-dsRNA into the cytoplasm, activates the PKR-eIF2α axis, triggers apoptosis and damage of renal tubular cells, and is associated with acute tubular necrosis, diabetic nephropathy, and other conditions.	1.Upregulate PNPT1 expression to promote mt-dsRNA degradation 2.Inhibit PKR activity to block eIF2α phosphorylation 3.Repair mitochondrial membrane permeability	107
	Diabetic nephropathy	mt-dsRNA release from mitochondria to the cytoplasm in DN, activating the PKR/eIF2α pathway and inducing tubular cell apoptosis.	1.inhibiting mt-dsRNA release 2.blocking PKR activation	108
Infectious disease​	Viral infection	Viruses induce abnormal accumulation of mt-dsRNA in cells, which are released into the cytoplasm via mitochondrial membrane channels or mitophagy pathways, activate MDA5/RIG-I, and trigger type I interferon antiviral immune responses.	1.Enhance mt-dsRNA degradation to avoid excessive immune activation 2.Regulate PRRs activity to balance antiviral responses	109
Cancer​	Cancer​	mtdsRNA that leaks into the cytoplasm is recognized by RIG-I, triggers a type I interferon response through the MAVS signaling axis, upregulates APOBEC3A in a STAT2-dependent manner, and thereby induces nuclear DNA damage. The mutational signature of the antiviral DNA deaminase APOBEC has been identified in over 70% of cancers.	1.Block RIG-I/MAVS/STAT2 signaling 2.Antagonize IFN-β secretion	112
	B-cell precursor acute lymphoblastic leukemia	mt-dsRNA serves as a pivotal trigger for the transformation of MSCs into cancer-associated fibroblasts CAFs.	1.Clear cytoplasmic mt-dsRNA through autophagy regulation	113
Cardiovascular disease	Atherosclerosis	mtRNA released into the cytoplasm activates PRRs and the NLRP3 inflammasome, exacerbates vascular inflammation, disrupts the endothelial barrier, and promotes monocyte infiltration and foam cell formation.	1.Clear cytoplasmic mtRNA to inhibit PRRs activation 2.Inhibit the NLRP3 inflammasome to reduce the release of proinflammatory cytokines	114, 115
Respiratory disease	Asthma	mt-dsRNA is recognized by PRRs (especially TLR3 and RLRs) to activate downstream signaling cascades, inducing the production of pro-inflammatory cytokines. Moreover, ETS2, which is upregulated in epithelial cells in asthma, can regulate the level of cytoplasmic mt-dsRNA through ANT2, thereby increasing cytokines such as IL-6, IL-5, and IL-13, and participating in the inflammatory process of asthma.	1.Targeted inhibition of PRRs 2. regulation of cytoplasmic mt-dsRNA levels 3.3inhibition of the upstream regulation of the ETS2-ANT2-mt-dsRNA axis.	116
